# Role of Selective Autophagy in Spermatogenesis and Male Fertility

**DOI:** 10.3390/cells9112523

**Published:** 2020-11-23

**Authors:** Chunyu Lv, Xiaoli Wang, Ying Guo, Shuiqiao Yuan

**Affiliations:** 1 Institute of Reproductive Health, Tongji Medical College, Huazhong University of Science and Technology, Wuhan 430030, China; chunyuwendy@163.com (C.L.); wangxiaoli@hust.edu.cn (X.W.); 2 Key Laboratory of Male Reproductive Health, National Health Commission of the People’s Republic of China, Beijing 100081, China; guoying0223@163.com; 3 Shenzhen Huazhong University of Science and Technology Research Institute, Shenzhen 518057, China

**Keywords:** autophagy, selective autophagy, spermatogenesis, male fertility

## Abstract

Autophagy is a “self-eating” process that engulfs cellular contents for their subsequent digestion in lysosomes to engage the metabolic need in response to starvation or environmental insults. According to the contents of degradation, autophagy can be divided into bulk autophagy (non-selective autophagy) and selective autophagy. Bulk autophagy degrades non-specific cytoplasmic materials in response to nutrient starvation while selective autophagy targets specific cargoes, such as damaged organelles, protein aggregates, and intracellular pathogens. Selective autophagy has been documented to relate to the reproductive processes, especially for the spermatogenesis, fertilization, and biosynthesis of testosterone. Although selective autophagy is vital in the field of reproduction, its role and the underlying mechanism have remained unclear. In this review, we focus on selective autophagy to discuss the recent advances in our understanding of the mechanism and role of selective autophagy on spermatogenesis and male fertility in mammals. Understanding the role of selective autophagy during spermatogenesis will promote the recognition of genetic regulation in male infertility, and shed light on therapies of infertile patients.

## 1. Introduction

Spermatogenesis is a complex biological process of germ cell proliferation and differentiation that produces a large number of spermatozoa in the seminiferous tubules. It contains three processes: mitosis of spermatogonia, meiosis of spermatocytes, and spermiogenesis, by which round spermatids transform to become the elongated spermatids [1]. Successful spermatogenesis, which progresses through precisely timed and highly organized cycles, is crucial to produce spermatozoa continuously and maintain adult male fertility. In contrast, abnormal spermatogenesis usually results in male subfertility or infertility [2]. The fine communication between germ cells and somatic cells within seminiferous tubules is fundamental to normal spermatogenesis. Among them, the support and nutritional function of Sertoli cells and endocrine function of Leydig cells are particularly important [3,4].

Autophagy is a highly conserved catabolic process for cell degradation, which is lysosomal dependent and essential for maintaining cellular homeostasis. The degraded components need to be sequestrated by double-membrane vesicles called autophagosomes, degraded by lysosomal enzymes after fusing with lysosomes, and finally complete autophagy [4]. In the beginning, autophagy was thought to be non-selective, which is called bulk autophagy. However, an increasing amount of evidence shows that autophagy can selectively degrade misfolded proteins and damaged organelles. The main factors involved in selective autophagy include autophagy receptors and adaptor proteins, which connect substrates to autophagy devices. According to the different substrates, selective autophagy can be divided into various subcategories, such as mitochondria (mitophagy), liposome (lipophagy), endoplasmic reticulum (reticulophagy), pathogens (xenophagy), peroxisomes (pexophagy), ribosomes (ribophagy), and aggregated proteins (aggrephagy) [5].

Recently, accumulative evidence shows that autophagy functions in a large number of cellular events during spermatogenesis, such as testosterone production, ectoplasmic specialization (ES) assembly, acrosome biogenesis, and cytoskeleton organization [6,7,8]. Both bulk autophagy and selective autophagy take part in various physiological processes, by degrading and recycling the cellular components, to ensure successful spermatogenesis [9]. Of note is that selective autophagy, which is involved in various membrane trafficking events, can maintain cellular homeostasis during spermatogenesis [5]. Although it has been reported in the literature that mitophagy, lipophagy, and reticulophagy are closely related to spermatogenesis [10,11,12], there is no effective and timely summary on the role of selective autophagy in spermatogenesis and male fertility. In this mini review, we will evaluate the current advances in our understanding of selective autophagy and discuss the underlying mechanism and mainly focus on the role of three selective autophagic processes (mitophagy, lipophagy, and reticulophagy) in spermatogenesis and male fertility. Our review will provide us with a better understanding of selective autophagy in spermatogenesis, which will help us broaden the diagnosis of spermatogenesis disorders and male infertility and identify more therapeutic targets for male infertility treatment.

## 2. Mitophagy

Mitochondrial homeostasis maintained by mitochondrial dynamics and mitophagy is important for the generation of energy, cellular homeostasis, steroidogenesis, and regulation of apoptosis [13]. Of note, mitophagy is a process that the cell selectively wraps and degrades damaged or superfluous mitochondria through autophagy, thereby maintaining mitochondrial homeostasis [14]. In addition, mitophagy has been implicated in the pathogenesis of cardiovascular disease and neurodegenerative diseases [15,16]. When mitochondria are damaged, mitochondria will split, and the damaged mitochondrion will be cleared by mitophagy to maintain the normal function of the mitochondria [13]. Thus, mitophagy could help to produce a new smaller healthy mitochondrion that is essential to the recycling of mitochondria function.

**Figure 1:**
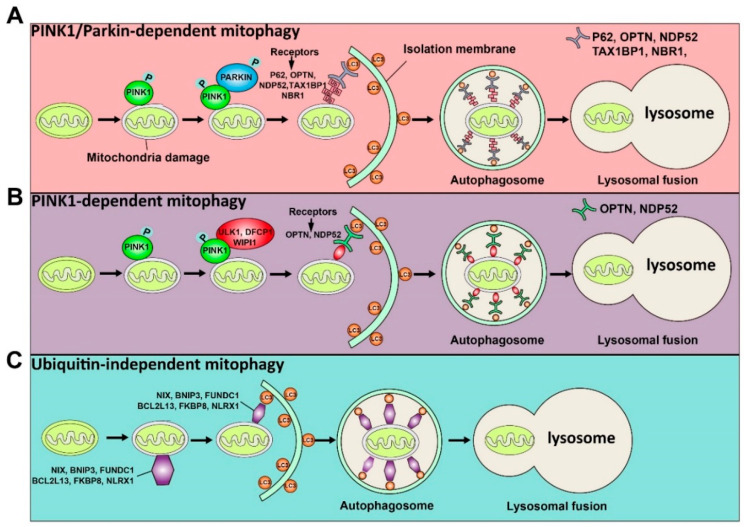
Schematic illustration of the ubiquitin-dependent and ubiquitin-independent pathways of mitophagy is shown. The ubiquitin-dependent mitophagy can be divided into PINK1/Parkin-dependent mitophagy (**A**) and PINK1-dependent mitophagy (**B**). (**A**) Once mitochondria are damaged, PINK1 will accumulate on the OMM (outer mitochondrial membrane) and activate it by phosphorylation. The activated PINK1 recruits and activates PARKIN by phosphorylation. Then, the activated PARKIN produces the polyubiquitin chains recognized by receptors (P62, OPTN, NDP52, TAX1BP1, and NBR1). These receptors will bind with the LC3 adaptor to engulf mitochondria to complete autophagy. (**B**) The activated PINK1 recruits ULK1, DFCP1, and WIPI1, which can be recognized by receptors (OPTN and NDP52) to induce PINK1-dependent mitophagy. (**C**) Some proteins, including NIX, BNIP3, FUNDC1, BCL2L13, FKBP8, and NLRX1, can directly recognize the LC3 adaptor and initiate the mitophagy process.

Although it has been reported that mitochondria can be degraded by bulk autophagy, the mechanism of mitophagy and bulk autophagy is different. Usually, the mechanisms by which the LC3 adaptor recognizes mitochondrial proteins and induces mitophagy can be divided into ubiquitin-dependent and ubiquitin-independent mechanisms. The induction of mitophagy via ubiquitin-dependent mechanisms can be further mediated by the PINK1 (a mitochondrial serine/threonine-protein kinase)/Parkin (a cytosolic E3 ligase)-dependent pathway or PINK1-dependent, Parkin-independent pathway [17]. PINK and Parkin were initially found to be associated with Parkinson’s disease [18]. Concretely, under normal circumstances, PINK1 can be imported into mitochondria through the translocase of the outer membrane (TOM) and the translocase of the inner membrane (TIM), so that the mitochondrial targeted sequence of PINK1 is cleaved by mitochondrial processing peptidase in the matrix, and PINK1 protein is degraded by protease presenilin-associated rhomboid-like protein (PARL) on the inner mitochondrial membrane (IMM) [19]. In contrast, when mitochondria are damaged, since the potential of the mitochondrial membrane decreases and the mitochondrial membrane depolarizes, PINK1 can only pass through the outer mitochondrial membrane (OMM), and PINK1 cannot enter the mitochondria to be degraded. At this time, PINK1 accumulates on OMM and is activated by phosphorylation [20]. The activated PINK1 phosphorylates ubiquitin at Ser65 to recruit and activate PARKIN ubiquitin ligase activity. Then, PARKIN produces polyubiquitin chains, which are recognized by autophagy receptors, including P62, OPTN, NDP52, TAX1BP1, and NBR1 [21]. Thus, receptor proteins will recruit mitochondria to the forming autophagosomes for degradation. Above is the PINK1/Parkin-dependent mitophagy pathway. In addition to the PINK1/Parkin-dependent mitophagy pathway, PINK1 can recruit OPTN and NDP52 to mitochondria in the absence of Parkin, and further recruit ULK1, DFCP1, and WIPI1 to induce the Parkin-independent mitophagy pathway [22]. Moreover, there are some proteins on the mitochondrial membrane that can directly recognize LC3 and induce mitochondrial autophagy directly, including NIX, BNIP3, FUNDC1, BCL2L13, FKBP8, and NLRX1 [22]. Among them, NIX and BNIP3 interact with LC3 through their BH3 domain, further inducing mitophagy [23,24], while FUNDC1 directly binds to LC3 to induce mitochondrial autophagy under hypoxic conditions [25]. BCL2L13, FKBP8, and NLRX1 directly bind to LC3 through their LIR motifs and induce mitophagy [26,27,28]. The ubiquitin-dependent and ubiquitin-independent pathways of mitophagy are illustrated in Figure 1. Importantly, it has been shown that defective mitophagy impairs spermatogenesis as discussed below.

### 2.1. Role of Mitophagy in Spermatogenesis

**Figure 2:**
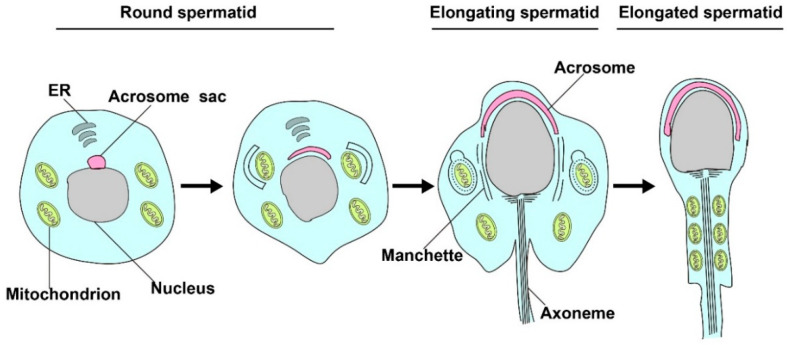
Mitophagy is involved in spermatid differentiation. In the process of spermiogenesis, excessive mitochondrion in the residual bodies will be cleared by mitophagy, and the remaining mitochondrion will rearrange in elongated tubules and participate in the formation of spermatozoa.

The late stage of spermatogenesis needs to undergo sperm transformation, also termed spermiogenesis, which requires sperm nucleus elongation and condensation, acrosome biosynthesis, and flagella formation. During this process, the removal of excess mitochondria in the residual bodies by mitophagy is key to produce individual spermatozoa and proper mitochondria rearrangement [29,30,31] (illustrated in Figure 2). Based on current literature, some autophagy-related proteins have been reported to play an essential role in spermiogenesis, as discussed below (also summarized in Table 1).

For instance, FBXO7, a receptor for SCF-type ubiquitin E3 ligase complex, participates in the PINK1/Parkin-dependent mitophagy pathway. FBXO7 acts downstream of PINK1 and depends on PINK1′s phosphorylation to recruit Parkin cooperatively to mitochondria, thereby inducing mitophagy [38]. Likewise, deficiency of *FBXO7* expression also blocks the translocation of Parkin to mitochondria and mitophagy [38]. In *Fbxo7*-deficient spermatids (*Fbxo7^LacZ/LacZ^* mice), nuclear elongation and replacement of histones by transition proteins are normal, but spermatids’ cytoplasm cannot be remodeled and eliminated correctly, which leads to spermatid death through wrongful phagocytotic cell processes [32]. Similarly, the Drosophila ortholog of *Fbxo7* is Nutcracker, and homozygous null-Nutcracker mutant spermatids cannot complete sperm individualization [39]. Moreover, in the Parkin mutant Drosophila, late spermiogenesis was arrested due to spermatid individualization’s failure, with a specific defect of abnormal mitochondrial derivatives [33]. Similarly, the deletion of *Pacrg* in mice, a homolog of the human PARKIN-coregulated gene, resulted from abnormal formation of the mid-piece of the sperm flagellum and improper removal of redundant cytoplasm, thereby leading to male sterility [40]. In male germ cell conditional knockout *Atg7* mice, the sperm flagella displayed severely coiled morphology with mislocalized and poorly condensed mitochondria, which caused the mitochondria rearrangement disruption and sperm motility decrease, further resulting in male infertility [6]. Likewise, ATG5 deficiency in male germ cells led to male subfertility with abnormal sperm differentiation, including aberrant acrosome biogenesis, enlarged residual bodies, and mitochondrial rearrangement, which is the result of disruption of autophagy activity [31]. In addition, the activity of AMPK (5’-AMP-activated protein kinase catalytic subunit α2), which is a AMP-activated protein kinase, is essential for sperm motility, the integrity of sperm membranes, and the mitochondrial membrane potential (ΔΨm) [41]. AMPK is located on the midpiece of mammalian sperm [42]. Inactivation of sperm AMPK will decrease the potential of sperm mitochondrial membrane, which is an important signal for mitophagy, and affect sperm motility [41,43]. These published data suggest that a deficiency of autophagy-related proteins fails to eliminate the cytoplasm of spermatid and individualize spermatid by rearrangement of mitochondria.

### 2.2. Role of Mitophagy in Post-Fertilization

Considering that the goal of spermatogenesis is to produce functional mature spermatozoa, the formation of functional spermatozoa is a vital link in the process of fertilization. Interestingly, sperm mitochondria DNA (mtDNA) needs to be cleared by mitophagy after fertilization but the maternal mitochondrial DNA retained and passed on to the next generation [44]. In *C. elegans*, the membrane organelles (MOs) of the sperm can be ubiquitinated after the sperm component enters the cytoplasm of the oocyte, which is marked by ATG8/LC3 ubiquitin-like proteins LGG-1/LGG-2 (homolog of GABARAP and LC3, respectively). Subsequently, the LGG-1/LGG-2 signal surrounds MOs and paternal mitochondria on account of close contact between sperm mitochondria with the ubiquitinated MOs, and the autophagy receptor further recognizes the components of the ubiquitinated label together and binds to the LC3-interacting region (LIR). Finally, the autophagosome engulfs these components and degrades them after combining with the lysosome to complete mitophagy [44]. In comparison, in Drosophila melanogaster, paternal mitochondrial can be cleared by the Parkin-independent mitophagy pathway [45]. Similarly, in mammals, the ubiquitination of sperm mitochondria is the signal for autophagy degradation [46]. The elimination of paternal mitochondria is also dependent on a mechanism involving ubiquitination and the lysosomal pathway [47]. Rojansky et al. identified that the E3 ubiquitin ligases Parkin and MUL1 coordinately function in the degradation of paternal mitochondria in the early mouse embryo [34]. Concretely, the mitochondrial membrane’s potential decreases after entering the oocyte, and the depolarization of the mitochondrial membrane would activate PINK1/Parkin-dependent mitophagy to eliminate paternal mitochondria [34]. Moreover, Rojansky et al. also found that more than half of double-knockout Parkin and MUL1 embryos still retained paternal mitochondria at 84 h after fertilization, while knocking out a single gene nearly did not retain the paternal mitochondria [34]. These results indicate that in the early mouse embryo, Parkin and MUL1 have a redundant function in mitophagy. However, the concrete mechanism remains be explored. In addition, there is clear evidence showing that autophagy inactivation will lead to the inheritance of paternal mitochondrial genes during the early embryo stage and heteroplasmy establishment [48]. A recent study showed that the heteroplasmy of mtDNA affects embryo metabolism and cell adaptability, which eventually affects embryonic development [49]. Furthermore, the persistence of paternal mtDNA may spread the potentially deleterious mitochondria to the whole body [44]. Taken together, all published evidence suggests that mitophagy plays an essential role in the process of sperm differentiation before fertilization and removal of paternal mitochondria after fertilization. Therefore, based on the published literature, we speculate that LC3 adaptor protein recognizes ubiquitinated sperm mitochondrial DNA after fertilization through the ubiquitin-dependent mechanism to achieve mitophagy. However, due to the lack of relevant studies, the specific pathway of mitophagy regulation in post-fertilization is still unclear.

### 2.3. Mitophagy and Male Infertility

From a clinical perspective, mitophagy is closely related to cryptorchidism and asthenozoospermia. A very recent study reported that the higher temperature in the body cavity not only causes spermatogenesis arrest of cryptorchidism but also induces damage of mitochondria and the initiation of mitophagy [43]. Simultaneously, during sperm transport to the epididymis, the mitochondrial outer membrane matures, mitochondria become an onion-like structure and undergo a series of modifications of their structure and localization in the cell, and finally serve as an energy provider for sperm motility. However, this cannot succeed due to cryptorchidism patients accompanied by epididymal anomalies, thereby causing the occurrence of asthenozoospermia [50]. Besides, mitochondria are the only organelle for sperm to produce reactive oxygen species [51]. Offensive production of reactive oxygen species can initiate mitophagy and cause male gamete to have an apoptosis-like phenotype [43]. In addition, mitophagy will be activated in Sertoli cells of acute ethanol-treated rats and has an antiapoptotic role for Sertoli cells [52]. However, the more in-depth studies about concrete mechanisms controlling mitophagy in Sertoli cells might have therapeutic significance for male infertility. Furthermore, an in-depth study of the relationship between mitophagy and spermatogenesis-related diseases will provide great clinical significance for the diagnosis and treatment of male infertility in the future.

## 3. Lipophagy

The lipid droplets (LDs), which are mainly made up of cholesteryl ester and triglycerides, are the main lipid storage form in living organisms. Its degradation can regulate the process of lipid metabolism to provide energy for cells. There are two main catabolic pathways to degrade LDs in response to nutrient limitation: lipolysis and lipophagy [53]. Lipolysis needs a large number of LD-related lipases to release lipids from LDs, the initiation of which needs adipose triglyceride lipase (ATGL) [54]. However, lipophagy, a process that releases fatty acids from LDs by autophagy, has more significant lipophagic activity during starvation compared to lipolysis [54,55]. In addition, lipophagy includes macrolipophagy and microlipophagy. It has been reported that SQSTM1/p62 (sequestosome-1/p62) protein is the key receptor for the specific recognition of LDs during macrolipophagy [56]. Interestingly, LDs can be recognized as a selective substrate and sequestered by the autophagosome, and degraded by hydrolase after combining with the lysosome, while in microlipophagy in yeast, LDs contact the vacuole (lysosome) directly with docking sites instead of being engulfed by the autophagosome [57]. Lipophagy is associated with fatty liver disease, obesity, renal cell carcinoma, and liver cancer cells [58,59]. Furthermore, lipophagy plays an essential role in energy metabolism and lipid homeostasis, and is not only closely related to hepatic diseases but also participates in the regulation of spermatogenesis [60]. We next focus on the understanding of lipophagy and summarize how lipophagy is involved in the regulation of spermatogenesis.

Lipophagy is regulated by many factors, such as Rab GTPase, transcription factors, hormones, and small molecules. For example, small Rab GTPase is involved in the regulation of fat-soluble proteins, and Rab GTPase can be used as a molecular switch cycling between active GTP and inactive GDP [61]. After nutrient depletion, the small GTPase on the LD surface will activate and switch to an active GTP state [62]. It is worth mentioning that this activated state will recruit the degradation devices (multivesicular bodies and lysosomes) to the vicinity of LD and degraded LD by lipophagy [63]. RAB protein is the most critical member of the Rab GTPase superfamily, and studies have found that Rab protein can affect lipid autophagy and metabolism [64,65]. Among them, RAB7 mainly participates in the process of autophagosomal maturation and intracellular transport [64] and functions in the fusion of autophagosome membranes and late endocytic membranes with the help of SNARE proteins and HOPS tethering complex [66]. Besides, it has been well established that RAB10 on the LD surface will be activated during starvation [65]. Additionally, Rab10 can promote the degradation of LD through lipophagy by interacting with EH-domain-binding protein 1 and membrane-deforming ATPase EHD2 [65]. Moreover, Rab10 knockdown results in increased LD accumulation in hepatocytes [65]. Additionally, RAB32 has been shown to co-localize with autophagy markers in Drosophila fat body [67]. In addition to RAB proteins, transcription factors can be involved in the regulation of lipid metabolism. For instance, transcriptional factor EB (TFEB) can induce lipophagy during lipid metabolism via the PPARα (Peroxisome proliferator-activated receptor alpha) and PGC1α (Peroxisome proliferator-activated receptor gamma coactivator 1alpha) signaling pathways [68]. Another transcriptional factor FOXO1 (Forkhead box protein O1) can trigger lipophagy by upregulating lysosomal acid lipase (LAL) and the autophagy gene Atg14 in adipocytes [69]. In addition, it is well known that the mTOR signaling pathway, which is involved in regulation of autophagy, can inhibit the initiation of autophagy and participate in the regulation of lipophagy as a modulator in response to the change of nutrients and hormones, such as glucose, amino acids, and insulin [15]. The activity of lipid metabolism and lipophagy will increase in rapamycin (mTOR signaling pathway inhibitor)-treated hepatocytes [70]. Interestingly, some small molecules can modulate lipophagy, such as caffeine, tetrandrine, the dietary polyphenol bergamot, and the red wine bioactive resveratrol [59].

### 3.1. Role of Lipophagy in Sertoli–Germ Cell Communication

**Figure 3:**
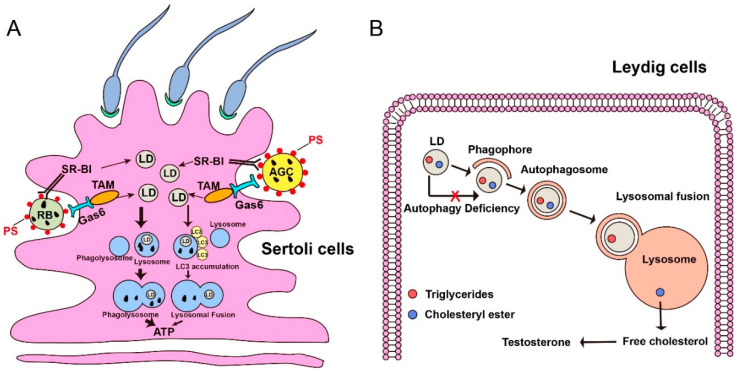
Lipophagy promotes spermatogenesis in Sertoli cells and regulates the synthesis of testosterone in Leydig cells. (**A**) During spermatogenesis, apoptotic germ cells (AGCs) and residual bodies (RBs) are phagocytosed by Sertoli cells by the SR-BI/PS (Class B scavenger receptor type I / Phosphatide) system or TAM/Gas 6 (Translocation and assembly module/Growth arrest-specific 6) system. The lipid droplets can release from the breakdown of engulfed AGCs and RBs. In this condition, most LDs fused with the lysosome to form phagolysosome to generate energy for Sertoli cells, which further supports germ cell and spermatogenesis. While, minor LDs can have an LC3 accumulation signal and then fuse with the lysosome to produce ATP for energy support. PS, a type of phospholipid. LD, lipid droplet. Bigger arrow, a major pathway to degrade LDs in Sertoli cells. Smaller arrow, a minor pathway to degrade LDs in Sertoli cells. (**B**) LDs are composed of cholesteryl ester and triglycerides, which are localized in the Leydig cells. In normal conditions, lipophagy can degrade cholesterol esters into free cholesterol, an essential substrate for testosterone synthesis. When autophagy deficient, the substrate of testosterone will decrease due to the failure of lipophagy.

Lipophagy within Sertoli cells can coordinate Sertoli–germ cell communication and provide energy for spermatogenesis [71]. However, the concrete mechanisms about how Sertoli cells provide energy for germ cells are not well understood. Based on the limited published literature [35,72], as discussed below, we speculate that lipophagy may be a good way to coordinate Sertoli–germ cell communication and provide energy for spermatogenesis. Firstly, in Sertoli cells of rats during the seminiferous epithelial cycle, LDs accumulated dramatically at stages IX-XIV, then decreased rapidly at stages I-III, and remained low at stages IV-VIII [73]. According to the distribution of LDs in different stages of the rat spermatogenic cycle, the decline at stages I-III can be attributed to the function of lipophagy, which was used for nourishing germ cells [71,73]. Moreover, the abundance of LDs at stages IX-XIV is derived from the re-synthesis of Sertoli cells [73], which provides a guarantee for the energy sources after spermiation. Thus, the degradation and re-synthesis of LDs that occur in the Sertoli cells may communicate well with germ cells. Secondly, an interesting study reported that there are two types of lipophagy to “eat” LDs within the Sertoli cells in the testes of the Chinese soft-shelled turtle [71]. One is that LDs were recognized and eaten into the autophagosome in the spring of May. Another is that LDs contact directly with autophagosomes and mitochondria in the autumn of October. In addition, the content and size of LDs within Sertoli cells at early spermatogenesis are much larger than late spermatogenesis [71]. This study indicates that as the spermatogenic cycle continues, the purpose of continuous consumption of LDs is to supply energy to developing germ cells continuously. After ejaculation in October, immature sperm are stored in the epididymis until the next mating due to the characteristic of seasonal mating of the Chinese soft-shelled turtle [74]. Before ejaculation, a small portion of the cytoplasm of spermatid remained, named the cytoplasmic droplet (CD). The release of LDs in the CD produces much fatty acid to ensure the long-term storage of spermatozoa in the epididymis [75]. Thirdly, the lipids broken from AGCs (apoptotic germ cells) and RBs (residual bodies) are the primary energy sources for Sertoli cells, which further provide physical and environmental support for spermatogenesis [76,77]. More than half of germ cells undergo apoptosis during spermatogenesis, and cytoplasmic portions of the elongating spermatids are shed as residual bodies (RBs) [78]. Once these cells undergo apoptosis, PS, a type of phospholipid, will translocate from the inner leaflet of the plasma membrane double layer to the outer leaflet of the cell membrane [79]. In this condition, AGCs and RBs are phagocytosed by Sertoli cells by the SR-BI/PS system and TAM/Gas6 system [77]. Then, LDs released from AGCs and RBs are mainly fused with the lysosome to produce ATP, which provides energy for Sertoli cells and supports germ cells [76,77,80]. In addition, the latest research found that autophagy is recruited to Sertoli cells for efficient clearance of AGC and RB and their degradation products via LC3-associated phagocytosis, as shown in Figure 3A [81]. The involvement of autophagy in the degradation of AGC and RB and LDs and communication between Sertoli cells and germ cells has attracted more and more researchers’ attention. In a word, the current literature suggests that lipophagy within Sertoli cells could promote spermatogenesis.

### 3.2. Lipophagy Regulates Synthesis of Testosterone

Not only can lipophagy within Sertoli cells promote spermatogenesis but lipophagy within Leydig cells can also support spermatogenesis by regulating the synthesis of testosterone [9]. Leydig cells located in the interstitial tissue of testes are important members of androgen production, especially testosterone. LDs, which are composed of cholesteryl ester and triglycerides, are the traits of Leydig cells. A previous study demonstrated that lipophagy, when it occurs in Leydig cells of the testes in the Chinese soft-shelled turtle, can degrade cholesterol esters into free cholesterol, an important substrate for testosterone synthesis [60]. As we all know, testosterone is essential for normal spermatogenesis and sexual development. In recent years, more and more studies have paid attention to the internal connection between lipophagy and testosterone production. Indeed, an autophagic deficiency is associated with reduced testosterone in aged rat Leydig cells [72]. In Leydig cells from young and aged rats, the level of luteinizing hormone(LH)-stimulated steroidogenic acute regulatory(StAR) protein and testosterone production decreased after dealing with Wortmannin (an autophagy inhibitor) [72]. Interestingly, this phenomenon can be rescued in Leydig cells from aged rats after dealing with rapamycin (an autophagy activator) [72]. Besides, androgen deficiency would mainly cause late-onset hypogonadism (LOH), which is the leading cause of abnormal male sex characteristics [82].

Furthermore, autophagic absence decreases the level of testosterone and further blocks normal spermatogenic processes in nonbreeding male naked mole-rats (NMRs), which finally causes infertility [12]. In addition, steroidogenic cell-specific knockout of *Atg7* or *Atg5* will cause Na^+^/H^+^ exchanger regulatory factor 2 (NHERF2) to be accumulated in Leydig cells, a negative regulator of scavenger receptor class B, type I(SB-BI) [35]. Abundant NHERF2 decreases the expression level of SR-BI and further leads to an insufficient cholesterol supply, and eventually results in reduced testosterone synthesis [35]. Moreover, an early study revealed that in the model of rat azoospermia induced by an experiment, the normal spermatogenic process could be recovered by administering testosterone of a high concentration into the testes [83]. More importantly, a certain degree of antiapoptotic activity of testosterone can protect germ cells from apoptotic death [84]. Therefore, the published studies suggest that lipophagy within Leydig cells could promote the biosynthesis of testosterone and maintain established spermatogenesis (Figure 3B).

## 4. ER-Phagy or Reticulophagy

The endoplasmic reticulum (ER), as the largest organelle in cells, is the primary place for synthesis, folding, processing, trafficking of proteins, and lipid synthesis. The ER needs continuous renovation to maintain the optimal quality of intracellular proteins and the integrity of the organelle [85]. Once cells encounter a strong stimulus (nutrient limitation, Ca^2+^ metabolic imbalance, toxin exposure, and sustained oxidative stress stimulation), many protective mechanisms will start to function to restore cell hemostasis [86]. Among them, the initiation of endoplasmic reticulum stress (ERS) is a common and critical event [86]. With the emergence of ERS, the unfolded protein response (UPR) and ER-associated protein degradation (ERAD)-mediated ubiquitin-proteasome system (UPS) will firstly be activated to help the misfolded and unfolded proteins restore their normal structure, and eventually restore the homeostasis of ER [87]. However, when the stimuli exist for a long time or the strength of the stimuli is too strong, the above two reactions cannot function enough to remove the misfolded and unfolded proteins from the ER [86]. In this case, endoplasmic reticulum autophagy (ER-phagy) degrades the damaged ER into fragments, which are then reassembled into a new ER to restore the ER’s function [86,88]. On the other hand, due to the double-edged sword effect of autophagy, the continued existence of ER-phagy will cause programmed cell death [89]. ER-phagy functions in cancer and disease, for example, colorectal cancer and sensory neuropathy [90,91]. Several studies have found that ERS can affect the normal spermatogenic process and male fertility [10,36,37,92,93], which will be discussed below.

### 4.1. The Effects of ERS on Spermatogenesis and Male Fertility

A recent study reported that irreversible ERS was triggered in *TBC1D20*-deficient Sertoli cells of male mice. The emergence of irreversible ERS could result in G1/S arrest and excessive germ cell apoptosis, which may further contribute to the male infertility phenotype [36]. In recent years, researchers have found that the treatment of nonylphenol (NP) in primary cultured rat Sertoli cells could induce ERS in Sertoli cells and activate the ERS signaling pathway subsequently [37]. In contrast, continuous exposure of NP could result in dysregulated expression of ER-related proteins and prolong the persistence of ERS, eventually leading to massive apoptosis of Sertoli cells and male infertility [37]. However, the mechanism about the phenotype of abnormal testicular development and decreased male fertility caused by nonylphenol (NP), an environmental contaminant, is not very clear yet.

**Figure 4:**
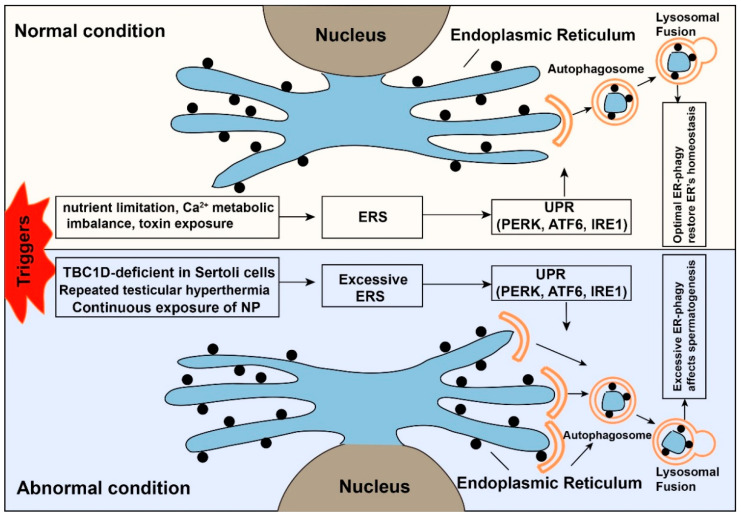
The dual role of endoplasmic reticulum autophagy. When cells face external stimuli, many protective mechanisms will be activated to restore the cells’ homeostasis. The upper panel shows endoplasmic reticulum stress (ERS) is a protective mechanism under normal conditions. The ubiquitin-proteasome system (UPS) and ER-phagy will be activated after the emergence of ERS. The normal function of ER and homeostasis of the cell will be restored. While the lower panel shows that under abnormal conditions, the intense or prolonged triggers (*TBC1D20*-deficient Sertoli cells of male mice, continuous exposure of nonylphenol, repeated testicular hyperthermia) will cause excessive ER-phagy, which affects the normal spermatogenic process.

In addition, heat-induced ERS mainly affects the normal spermatogenic process and male fertility by disrupting steroidogenic enzyme and testosterone production in Leydig cells [92]. Usually, in response to heat-induced ERS, the host cells initiate the UPR signaling pathway to restore the ER’s function. At the same time, repeated testicular hyperthermia would produce ERS continuously, thereby leading to apoptosis of Leydig cells, failure of spermatogenesis, and male infertility [92]. Similarly, the induction of excessive ERS in the Drosophila male accessory gland will cause the phenotype of infertility [10]. Thus, continuous persistence of ERS can damage the normal process of spermatogenesis and finally cause male infertility (Figure 4).

### 4.2. ERS Trigger Autophagy

A large number of studies have shown that ER-phagy is a novel way to relieve ERS for restoring the function of ER and homeostasis of cells [94,95]. The initiation of ER-phagy after ERS can be mediated through the UPR signaling pathway, releasing calcium ion into the cytoplasm [93]. Concretely, when cells encounter external stimuli, the UPR signaling pathway will be firstly initiated in response to ERS. At this time, the chaperone GRP78/Bip will escape from the three ER stress receptors (pancreatic ER kinase (PKR)-like ER kinase (PERK), activating transcription factor 6 (ATF6), and inositol-requiring enzyme 1 (IRE1)) and participate in the folding of abnormally accumulated proteins, and then PERK, IRE1, and ATF6 will start the respective reaction [96]. The activation of PERK blocks general protein synthesis by phosphorylating eukaryotic initiation factor 2α (eIF2α), thereby enabling the translation of ATF4, a transcription factor, which can induce ER-phagy by regulating some ATG genes [97]. However, a heightened degree/duration of ERS will trigger apoptosis by degrading the X-linked inhibitor of apoptosis protein (XIAP) and cooperating with C/EBP-homologous protein (CHOP/GADD153) [98]. Importantly, in the early stage of ERS, the IRE1-TRAF2-JNK pathway could activate ER-phagy to alleviate the swollen ER filled with misfolded and unfolded proteins. In contrast, JNK was activated continuously by prolonged ERS, which eventually led to apoptosis [88,99]. ATF6 was reported to translocate into the Golgi compartment after dissociating from GRP78 [100]. Besides, the cleaved active form of ATF6 can recognize some genes, such as GRP78, GRP94, transcription factors CHOP, and X box-binding protein 1 (XBP1) [97]. Thus, ATF6 might be involved in the regulation of ER-phagy indirectly due to the association with XBP1 and CHOP. Furthermore, ER is the only organelle containing high concentrations of calcium ion, and the calcium ions can also regulate ER and apoptosis under ERS [99]. On the one hand, releasing Ca^2+^ from the ER can inhibit mTOR and induce ER-phagy [99]. On the other hand, Ca^2+^ signaling can also promote apoptosis mainly by Ca^2+^-mediated mitochondrial cell death under excess ERS [99]. Thus, ER-phagy can promote the recovery of normal ER function when the ERS is within the adjustable range. While the level of ERS exceeds the normal load of ER, apoptosis will be triggered to cause cell death by weakening the function of ER-phagy.

Therefore, according to the degree of ERS, ER-phagy has dual functions, including pro-survival and pro-death. In other words, a certain degree of ERS can initiate ER-phagy to help remove abnormally folded proteins accumulated in the ER. In sum, ER-phagy might be involved in the regulation of ERS, which affects the normal spermatogenic process and male fertility. However, the concrete mechanism of how ERS triggers autophagy to regulate spermatogenesis still needs more experimental analyses to be further elaborated.

## 5. Therapeutic Modulation of Autophagy to Treat Male Infertility

Infertility is defined as failing to achieve a clinical pregnancy after 12 months or more of regular intercourse without contraception [101]. In recent years, infertility has become a global public health issue, affecting 15% of all reproductive-age couples. Among these, male factors account for ~25% of cases of infertility cases, especially abnormal semen quality [102,103]. More and more studies believe that the components of diet and nutrients may be an important factor affecting sperm quality and fertility [103,104]. For instance, diets rich in calories, trans-fatty acids (TFAs), saturated fats, or cholesterol have a harmful role in spermatogenesis and male fertility [105,106,107]. These bad diet habits often lead to obesity, related to impaired fertility [108,109]. Previous studies have demonstrated that autophagy was increased in obese individuals by feeding a high-fat diet (HFD) [110,111]. Jing Yang’s group investigated the role of autophagy in HFD-induced spermatogenesis deficiency [112]. They found that the level of autophagy increased in HFD mice, and spermatogenesis and male fertility were also disrupted in HFD mice. To know the correlation between autophagy and spermatogenesis deficiency in HFD mice, they inhibited and induced autophagy by injecting CQ and RAP intraperitoneally. They found that HFD mice subjected to CQ, an inhibitor of autophagy, showed improved spermatogenesis and decreased infertility. Simultaneously, autophagy was also overactivated in sperm samples from obese subfertile male patients [112]. Inhibition of excessive autophagy could protect against HFD-induced spermatogenesis deficiency and male infertility. This can provide a new clinical therapeutic method for increasing semen quality and male infertility.

## 6. Conclusions and Future Perspectives

Spermatogenesis is a dynamic complicated process that sustains mature spermatozoa production and underpins male fertility during the whole life of an adult male. In this mini review, we summarized and discussed the cumulative achievements, revealing that selective autophagy is active in many aspects of spermatogenic cycles; nevertheless, this is only the tip of the iceberg. We mainly discussed the function of three types of selective autophagy: (1) Mitophagy functions in the process of sperm differentiation before fertilization and helps remove paternal mitochondria DNA after fertilization. Previous papers have shown that most sperm mitochondrial DNA is removed by endonuclease G during spermatogenesis, subsequently leaving vacuolated mitochondria to participate in the formation mid-piece of spermatozoa. Additionally, energy produced by sperm mitochondria is also helpful for sperm motility and fertilization. In addition, the removal of paternal mitochondrial DNA after fertilization is conducive to embryo homogeneity and an individual’s health. However, the mechanisms of mitophagy in spermatogenesis and post-fertilization remain elusive. (2) Lipophagy promotes spermatogenesis and guarantees male fertility. In Sertoli cells, lipophagy can provide energy for germ cell development. The endocrinal function of Leydig cells promotes the biosynthesis of testosterone, thereby maintaining established spermatogenesis. (3) ER-phagy recovers normal cell homeostasis according to the extent of ERS. ER-phagy cannot rescue the normal function of the ER when the extent of ERS is too strong. More importantly, excessive ERS can also disrupt the normal process of spermatogenesis and decrease male fertility. Thus, the published research suggests that ER-phagy is involved in the regulation of ERS, which affects the normal spermatogenic function and male fertility. However, the concrete mechanism of selective autophagy in spermatogenesis remains to be elucidated. It is worth noting that there are still other types of selective autophagy, including xenophagy, pexophagy, and aggrephagy. For instance, xenophagy is a process in which the cell selectively removes intracellular microbial pathogens. While many pathogenic microorganisms pose a threat to spermatogenesis and male fertility, the concrete mechanism is still unknown. More importantly, autophagy also has a role in female fertility, including oogenesis, folliculogenesis, pregnancy, and pregnancy-associated diseases. However, the research of selective autophagy in female fertility is immature, especially the relationship between mitophagy, lipophagy, and ER-phagy, and female fertility still needs further exploration. Therefore, it is critical to address the roles of other types of selective autophagy in spermatogenesis and male fertility in the future.

## Figures and Tables

**Table 1:** Mutant animal models and the role of selective autophagy on spermatogenesis and male fertility in the main studies of this review.

Animal Models	Type of Autophagy	Phenotype	References	Correlation with Spermatogenesis
*Fbxo7* knockout in mouse germ cells	PINK1/Parkin-dependent mitophagy	Sterility	[32]	spermatids cytoplasm cannot be correctly remodeled and eliminated
*Parkin* null mutant in Drosophila	PINK1/Parkin-dependent mitophagy	Sterility	[33]	late spermiogenesis was arrested due to failure of spermatid individualization and abnormal mitochondrial derivatives
*Atg7* knockout in mouse germ cells	Mitophagy	Sterility	[6]	Abnormal Acrosome biogenesis
*Atg5* knockout in mouse germ cells	Mitophagy	Subfertility	[31]	Abnormal sperm differentiation and mitochondrial rearrangement
*Parkin* and *Mul1* knockdown in mouse embryo	PINK1/Parkin-dependent mitophagy	UN	[34]	Defect of degradation of paternal mitochondria in the early mouse embryo
In the nonbreeding male naked mole-rats (NMRs)	Lipophagy	Sterility	[12]	Reduced testosterone synthesis
*Atg5* or *Atg7* knockout in mouse Leydig cells	Lipophagy	UN	[35]	Reduced testosterone synthesis; Affect sexual behavior
*Tbc1d20* knockout in mouse Sertoli cells	ER-phagy	Sterility	[36]	Emergence of irreversible ERS; G1/S arrest and excessive germ cell apoptosis
Continuous exposure of Nonylphenol in primary cultured rat Sertoli cells	ER-phagy	Sterility	[37]	Massive apoptosis of Sertoli cells

UN: undetermined.
